# Caught Napping

**Published:** 1897-09

**Authors:** 


CAUGHT NAPPING.—A good joke on Love. Love is usually wide awake, but the burlesque prospectus of Weatherford College of Physicians and Surgeons caught him napping. He received the prospectus, and, without reading it (of course he didn't read it or he would have seen it was a take off), proceeded to inveigh, editorially, against the multiplication of colleges, in all seriousness. He did not observe that one of the qualifications for matriculation was that the applicant should be able to spell "Baker." Good joke on Love and the *Medical Mirror*.

